# Use It or Lose It: Facilitating the Use of Interactive Data Apps in Psychological Research Data Sharing

**DOI:** 10.5964/ejop.12811

**Published:** 2024-08-30

**Authors:** Franziska Usée, Christiane A. Melzig, Dirk Ostwald

**Affiliations:** 1 Department of Psychology, Clinical Psychology, Experimental Psychopathology, and Psychotherapy, Philipps-Universität Marburg, Marburg, Germany; 2 Center for Mind, Brain and Behavior, CMBB, Philipps-Universität Marburg and Justus Liebig University Giessen, Giessen, Germany; 3 Institute of Psychology, Research Methods, Otto von Guericke University Magdeburg, Magdeburg, Germany; Leibniz Institute for Psychology (ZPID), Trier, Germany

**Keywords:** open research data, data reuse, interactive data apps, innovative supplements, Dash, Gradio

## Abstract

The value of open research data (ORD), a key feature of open science, lies in their reuse. However, the mere online availability of ORD does not guarantee their reuse by other researchers. Specifically, previous meta-scientific research has indicated that the underutilization of ORD is related to barriers at the level of the ORD themselves, potential reusers of ORD, and the broader academic ecosystem. At the same time, sharing large datasets in an understandable and transparent format that motivates researchers to explore these datasets remains a fundamental challenge. With the present work, we propose interactive data apps (IDAs) as innovative ORD supplements that provide a means to lower barriers of ORD reuse. We demonstrate the use of two open-source Python libraries (Dash, Gradio) for IDA development using two psychological research use cases. The first use case pertains to an experimental quantitative dataset acquired in a clinical psychology setting. The second use case concerns the familiarization with data analysis workflows that are characteristic of natural language processing (NLP). For both use cases, we provide easy-to-adapt Python code that can form the basis for IDA development in similar scenarios.

We live in a digital era in which scientific progress is largely fueled by the availability and use of data that are rich in volume, velocity, and variety. Data thus represents a highly valuable resource that – like oil – must be refined and published at a high quality to make full use of it (Economist, 2017). Large amounts of public funds are spent annually on research data acquisition and knowledge generation. However, science and society only benefit the most from these investments, if these data are available as *open research data* (ORD; UNESCO, 2021). That is, ideally, any form of scientific information that is used as evidence for a phenomenon as well as scientific metadata, protocols, analysis source code, and workflows should be openly accessible in a human- and machine-readable format (Borgman, 2017; Molloy, 2011). It is by now generally accepted that the responsible use of scientific resources requires researchers to ensure that their data are sufficiently findable, accessible, interoperable, and reusable, that is, that their data are *FAIR* (Mons, 2018; Wilkinson et al., 2016).

The primary objective of FAIR data is ORD reuse (Joo et al., 2017; Molloy, 2011; Wilkinson et al., 2016). Following Pasquetto et al. (2017), we understand ORD reuse as any secondary ORD use by researchers other than the data originators, often in pursuit of a different purpose than the one intended in the original study (Faniel & Jacobsen, 2010). In fact, working with data collected by fellow researchers affords numerous advantages for the academic enterprise. First, the time and financial costs associated with data acquisition are eliminated, resulting in faster and more efficient research cycles (Curty & Qin, 2014; Kim & Yoon, 2017; Quarati & Raffaghelli, 2022). When researchers are freed from data acquisition duties, they are theoretically able to spend more time and intellectual capacity on sophisticated data analysis and careful interpretation of statistical results, potentially enhancing the overall quality of their research. Second, ORD reuse facilitates the verification, validation, and replication of scientific results, thus reducing the aversive effects of questionable research practices (Hagger, 2022). Third, combining ORD from multiple sources allows new and long-standing research questions to be addressed in a more powerful and, not least, more ethical manner (Brakewood & Poldrack, 2013). Generally speaking, ORD reuse thus promotes the economic use of scarce public resources and sustainable research.

However, despite its multifaceted and well-known benefits, ORD reuse is not standard academic practice and many openly available research datasets remain underutilized (Quarati & Raffaghelli, 2022). For example, in 2019, only half of more than 8,400 surveyed international academics stated that they had used ORD (Digital Science et al., 2019). Similarly, Imker et al. (2021) found that only approximately one-third of those researchers who generated and shared ORD reported that the data were actually reused. Moreover, studies on the qualitative aspects of researchers’ data reuse behavior indicate that if ORD is in fact reused, it is most often employed for comparative purposes, as a baseline measurement, or for calibration purposes, but not for addressing novel original research questions or applying new data analytical workflows (Pasquetto et al., 2019; Wallis et al., 2013). At least three reasons for the underutilization of ORD have recently been investigated. These can be broadly categorized as pertaining to the ORD state itself, potential reusers of ORD, and the broader academic ecosystem in which ORD generation and reuse take place.

First, ORD may not be sufficiently accessible for humans’ active engagement. In this context, it has been repeatedly shown that poorly curated data as well as restrictions on data access and data (re-)usage negatively influence data reuse behavior (Molloy, 2011; Ouzzani et al., 2013). Because ORD reuse requires trust in other people’s work, low ORD curation standards may induce concerns regarding data quality and the necessary time investments for using data productively (Imker et al., 2021; Pasquetto et al., 2019; Quarati & Raffaghelli, 2022; Tenopir et al., 2015; Wang et al., 2021). Moreover, ORD reuse intentions have been found to be strongly correlated with the perceived usefulness of ORD (e.g., increases in research productivity; Joo et al., 2017; Kim & Yoon, 2017; Yoon & Kim, 2017). Second, ORD reuse demands certain levels of data literacy by potential data reusers (Pasquetto et al., 2019; Quarati & Raffaghelli, 2022). As data literacy remains an educational challenge across academic disciplines (Raffaghelli & Sangrà, 2023), there is evidence of a lack of knowledge concerning the accessibility of ORD as well as the technical skills to engage effectively with ORD. For instance, researchers interviewed as part of a study by Suhr et al. (2020) mostly reported a lack of familiarity with the concept of dataset search engines such as Google’s Dataset Search. Furthermore, researchers might be afraid of the time and intellectual resources required to make use of ORD, especially when they lack sufficient digital and programming skills (Suhr et al., 2020). Third, in most research communities, ORD generation and reuse are rarely encouraged and rewarded. Unsurprisingly, most researchers report that ORD reuse is only “somewhat encouraged” by their co-workers, their research community, or their organization and most researchers do not feel sufficiently informed, motivated, and supported to generate and reuse ORD (Digital Science et al., 2022; Gregory, 2020). Overall, there is ample evidence that the mere online availability of ORD does not guarantee their reuse, which calls upon researchers and institutions alike to undertake additional steps to maximally benefit from the added value of ORD.

A potential means to increase ORD reuse are *interactive data apps* (IDAs) that accompany primary research artifacts such as journal articles as innovative supplements. An IDA may be defined as any form of web-based application that facilitates ORD accessibility by serving the human information-seeking mantra of “overview, zoom, filter, details on demand” (Murray, 2017; Shneiderman, 1996; Ward et al., 2010). Popular and familiar IDAs include the US National Weather Service Dashboard (https://weather.gov; National Research Council, 1999), the Johns Hopkins University Center for Systems Science and Engineering COVID-19 Dashboard (https://coronavirus.jhu.edu/; Dong et al., 2022), and the Twitter/X hedonometer (https://hedonometer.org/; Dodds et al., 2011). While IDAs are becoming increasingly popular in healthcare settings, where they are leveraged to support data-based decision-making by reducing cognitive demands for clinicians (Almasi et al., 2023; Bucalon et al., 2022; Khairat et al., 2018), and as educational tools (Fawcett, 2018; Jiang et al., 2022), the use of IDAs in psychological ORD generation and reuse remains underdeveloped.

This is surprising because IDAs afford the potential to make psychological ORD more accessible and attractive by directly targeting the identified reasons for ORD underutilization. First, with regard to ORD curation, developing IDAs as part of a primary research communication requires a high level of attention to data curation because only well-formatted and curated data can form an effective basis for the automated data processing that IDAs implement. Second, and perhaps most importantly, regarding potential ORD reusers, IDAs can lower the threshold of engaging with ORD because IDAs offer an intuitive, interactive, and non-technical approach to easily understand a dataset’s key features and reuse capacity. Finally, a more widespread adoption of IDAs within the academic community has the potential to render IDAs a standard companion of primary research artifacts with the potential to induce a systemic change towards higher levels of ORD generation and reuse both within the academic community and beyond.

With the present work, we aim to facilitate the use of IDAs in psychological research data sharing by providing a tutorial introduction to IDA development as an innovative supplement for ORD in psychology. After introducing basic steps to consider in the development of IDAs for ORD, we discuss two practical use cases. The first use case pertains to an experimental quantitative dataset acquired in a clinical psychology setting. The second use case concerns the familiarization with data analysis workflows that are characteristic of natural language processing (NLP). For both use cases, we use an individual Python-based application framework and provide easy-to-adapt Python code that can form the basis for IDA development in similar scenarios.

## IDA Development

The development of IDAs is currently supported by several programming languages. We focus on Python due to its general-purpose nature, high readability, and widespread use (Stack Overflow Developer Survey 2023; for IDA development using R/Shiny, see Ellis & Merdian, 2015 and for IDA development using R/Dash, see https://dash.plotly.com/r). In general, IDA development for data in the format of ORD can be structured into three basic steps:

1. Definition of the IDA’s aims and scope,
2. IDA programming and testing, and
3. Deployment of the IDA for web-based presentation.

In the following, we will discuss these steps in greater detail by means of two practical use cases that were developed using two open-source Python libraries, namely, *Dash* (Plotly Technologies Inc; https://plotly.com/dash/) and *Gradio* (Abid et al., 2019; https://www.gradio.app/). Each Python library was chosen based on its relative popularity and applicability to the use case at hand. To follow along with the examples below and execute or adapt the accompanying Python code on a local machine, Python (https://www.python.org), a suitable integrated development environment (IDE) such as Anaconda Spyder (https://anaconda.org/anaconda/spyder) or Visual Studio Code (https://code.visualstudio.com/), and the required Python libraries must be installed (e.g., using *pip install -r requirements.txt*).

## Development of a Data Exploration Board Using Dash

### Introduction to Dash

Dash is an open-source Python library that allows for the rapid development and deployment of web applications. It is built on top of Flask, a minimalistic web application framework (https://flask.palletsprojects.com/), and two JavaScript libraries, React.js (https://react.dev/) and Plotly.js (https://plotly.com/javascript/) for interactive visualization. IDA development with Dash itself requires no knowledge of JavaScript. From the user perspective, Dash offers a high degree of IDA design flexibility while simultaneously being of low-code design, meaning that minimal Dash applications can be built with a few lines of Python code. Due to its high IDA design flexibility, the application areas of Dash-based IDAs are diverse and range from data exploration to interactive knowledge transfer. The Dash Python library itself consists of several modules, each addressing certain aspects of IDA development, such as IDA layout design or the facilitation of interactive IDA features. Due to its origin, visualizations are most commonly and easily implemented using the Python Plotly Graphing Library (https://plotly.com/python/) which provides access to a wide range of chart types.

**Figure 1:**
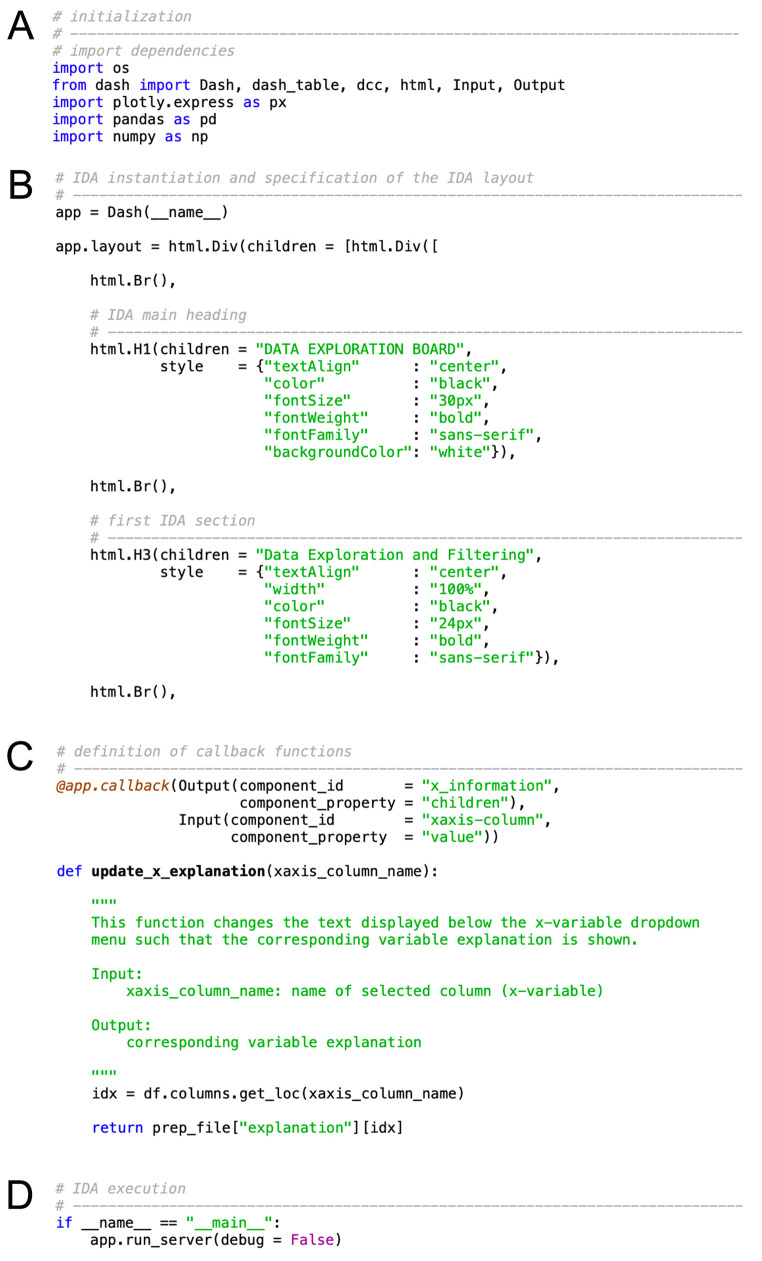
General Structure of Python-Based Dash Scripts

The general structure of Python-based Dash scripts is displayed in Figure 1. First, the required dash modules and additional Python libraries are imported (see Figure 1A). Second, the IDA is instantiated by the so-called Dash constructor *app = Dash(__name__)*, and the layout of the IDA is specified in terms of the components to be displayed in the web application (see Figure 1B). Accordingly, this part will vary the most between different IDAs. The building blocks for the layout customization are Dash Hypertext Markup Language (*html*) components and Dash core components (*dcc*). Whereas the former allow for the simple conversion of Python syntax to HTML syntax (e.g., for the definition of a line break, *html.Br()* is converted to *<br*>), the latter provide access to interactive elements, such as dropdown menus, sliders, and upload or download buttons. The layout is most commonly defined as a list of these components, usually embedded in a container element specified by means of *html.Div()*. Each container element consists of a *children* and a *style* argument that define content and presentation aspects of the component, respectively. Style attributes are usually specified using camelCase, such as *fontSize* or *marginLeft*. Important aspects to consider when customizing the IDA layout are the ordering of elements, as components will be displayed in the order they are defined, and the definition of component identifiers for interactivity purposes via the *id* argument.

The third component of a Dash script is the definition of callback functions that provide the basis for IDA interactivity (see Figure 1C). Callbacks are functions that are automatically executed whenever changes to the values of their input components are made. In response to these changes, callback functions update properties of one or more output IDA components. Thus, each callback function consists of at least one output component, one input component, and a function definition that specifies the changes to be made to the output components in response to changes of the input components. Both output and input components must be defined in terms of their component identifier (*component_id*) and their value of interest (*component_property*). Notably, the number of callback function arguments must be equal to the number of listed components within the callback initialization *(@app.callback()*). While the names of the function arguments can be freely chosen, the order is predetermined by the callback initialization.

Finally, a Python-based Dash script executes the IDA, with the *debug* argument specifying whether additional developer tools for debugging should be activated (see Figure 1D). By default, the IDA is run locally. For sharing purposes, additional web hosting services such as servers must be employed (e.g., by using public server services on the web).

### Use Case 1: A Data Exploration Board

In our first use case, we consider the development of an IDA for the common scenario of ORD stemming from a small-scale experimental psychology laboratory study in the domain of clinical psychology. The IDA can be accessed directly using a standard web browser at http://dashida.pythonanywhere.com/. It is based on research data comprising behavioral measures, self-report measures, and sociodemographic measures from 50 adults who participated in an experiment on approach-avoidance behavior (*n* = 33 female, *M*_Age_ = 22.98 years, *SD*_Age_ = 3.62).

In brief, the experiment consisted of three experimental phases (*learning phase, test phase, extinction phase*) comprising 92 experimental trials in total. On each trial, participants were asked to forage one of four possible visual search field configurations (*patches*) by clicking on targets presented within the field. Each search field configuration represented an experimental condition in a 2 x 2 factorial design with the within-subject experimental factors *gain* (positive reward) and *punishment* (negative reward). Specifically, each visual search field contained a condition-specific number of hidden coins to forage (low positive reward: two coins, high positive reward: six coins) and was associated with a condition-specific probability of receiving an electrical shock (low negative reward: probability of shock .1, high negative reward: probability of shock .8). In the learning phase of the experiment, participants were asked to learn these unknown associations between search fields and experimental conditions by actively engaging within the experimental environment. In the subsequent test phase, participants were allowed to make use of their acquired knowledge by actively deciding when to stop foraging a given search field. Finally, in the extinction phase, no electrical shocks were presented while the participants were asked to search for hidden coins, facilitating the unlearning of the aversive stimulus condition.

As the basis for the development of the IDA, the raw data were preprocessed, including, but not limited to, the detection and handling of missing values and outliers, the computation of condition-specific behavioral measures, and the calculation of self-report questionnaire scores. Finally, the preprocessed data were aggregated on the levels of participants (*n* = 50), experimental phases (*n* = 3), and experimental conditions (*n* = 4). In total, the dataset contains 596 data entries for 48 variables of interest (data from the extinction phase is missing for one participant).

#### Definition of the IDA’s Aims and Scope

**Figure 2:**
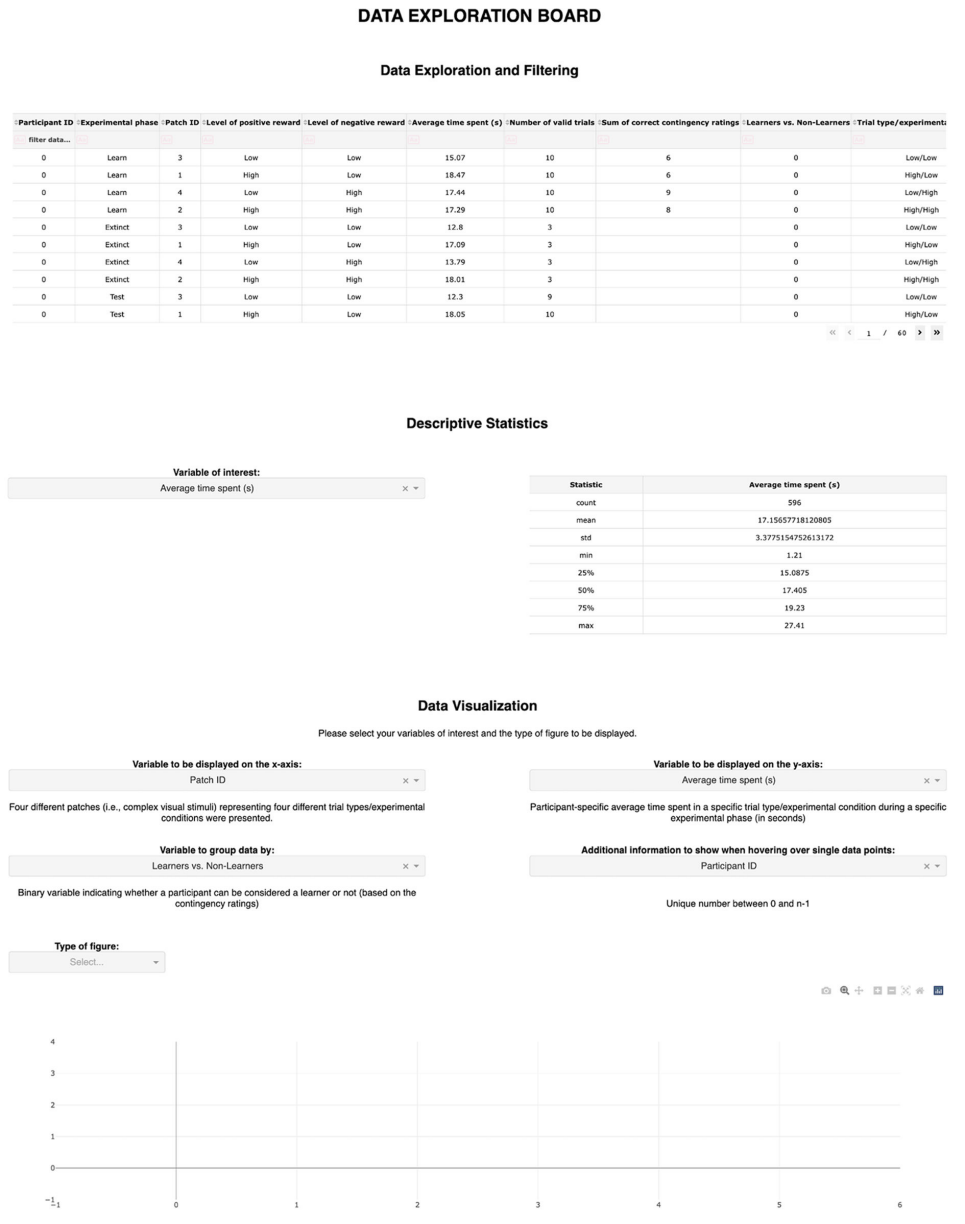
A Data Exploration Board

For the ORD of the current use case, the IDA aims to facilitate data exploration and familiarization for potential dataset reusers, such as other researchers studying approach-avoidance behavior. As the ORD stems from a rather complex experimental paradigm including many variables from different data sources, we reasoned that the mere sharing of the data in the form of a static data table may limit both the understanding of the key data features as well as the attractiveness of the dataset. With the current IDA, we thus aimed to provide an interactive, informative, and joyful experience when exploring the data. To achieve our aims in the spirit of the human information-seeking mantra (Murray, 2017; Shneiderman, 1996; Ward et al., 2010), we wanted IDA users to be able to interactively overview, sort, and filter the data, inspect commonly recommended descriptive statistics of all key variables (Gravetter et al., 2021), and visualize filtered data subsets of interest (see Figure 2).

#### IDA Programming and Testing

Based on the defined IDA’s aims and scope, the IDA layout was specified in terms of three sections, including an interactive data table, the presentation of descriptive statistics, and user-generated two-dimensional data visualizations. To enhance the understandability of variable names, we harnessed an additional data file (*prep_file.csv*) with information on variables to be displayed within the IDA, including their original names as specified in the ORD, the names to be used within the IDA environment, variable explanations, and information on specific variable types and transformations to be considered, such as whether a variable should be represented as categorical. By means of this data file, the ORD could be left unchanged in its original format and all data modifications necessary for presentation within the IDA could be restricted to the Dash script.

For the IDA’s layout, we mainly used HTML and Dash core components to implement, for instance, the various dropdown menus and graphs. The side-by-side arrangement of certain IDA components was achieved by setting the *display style* argument to *inline-block*. To introduce the desired interactivity to the IDA, multiple callback functions were defined. Specifically, these callback functions implement the updating of information displayed below dropdown menus in response to changes made to the corresponding dropdown menu, the modification of descriptive statistics in response to changes made to the corresponding dropdown menu and changes of data filtering settings, as well as the generation of graphs in response to changes made to the dropdown menus within the data visualization section and changes of data filtering settings. When the IDA is first loaded, no data visualizations are displayed and users are required to actively select variables and graph types, such as scatter or line plots, for visualization. Note that all filtering settings applied to the data table are always respected by the subsequent IDA sections.

**Figure 3:**
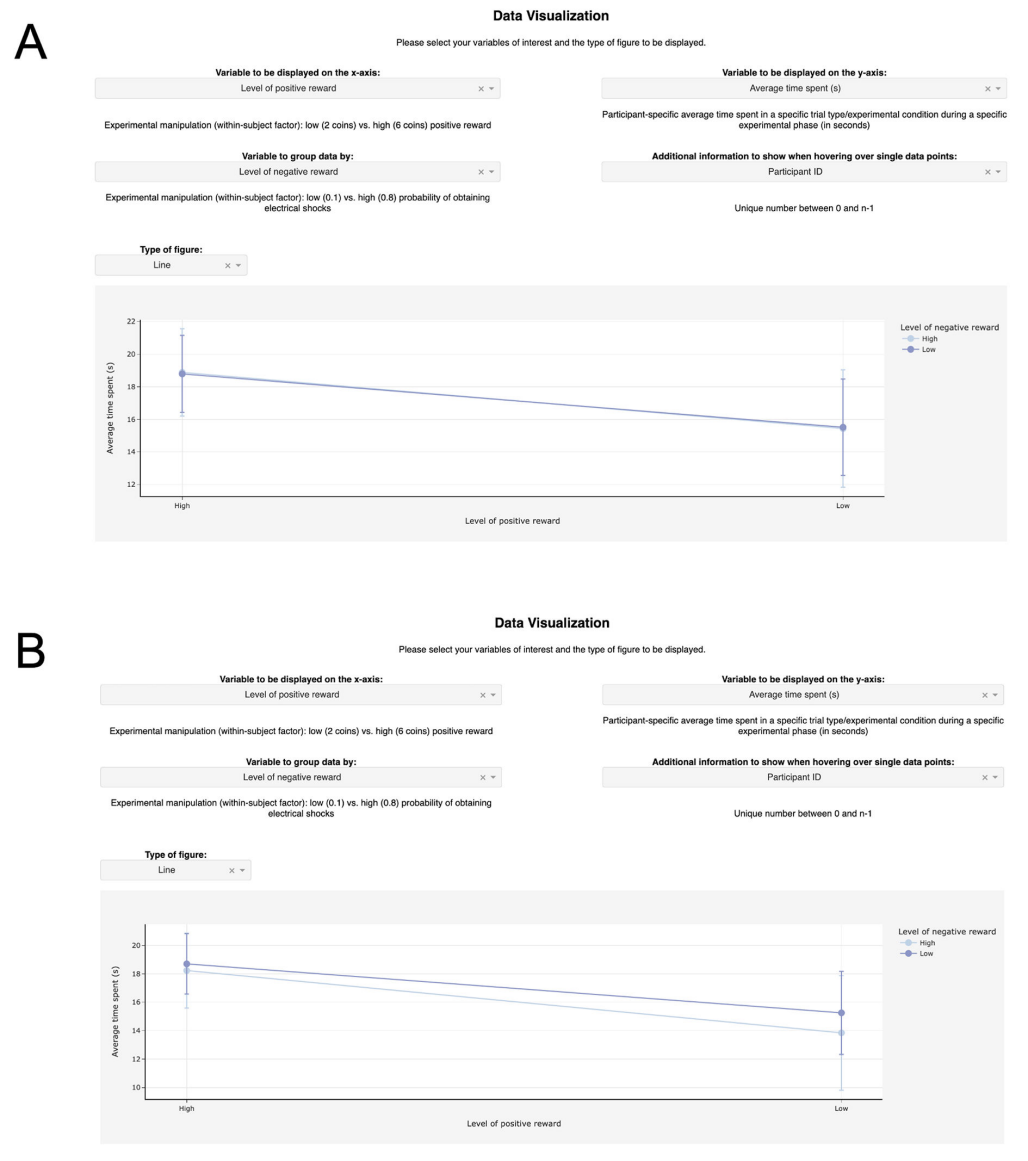
Demonstration of the Influence of Data Filtering Settings *Note.* A: Line plot generated by using all data entries. B: Line plot generated by filtering the data table for learning phase entries.

As an example of the IDA’s interactivity, Figure 3A displays a line plot generated by using all data entries, i.e., no data filtering, whereas Figure 3B displays the same line plot generated by filtering the data table for learning phase entries only, which can be achieved by typing *Learn* below the variable name *Experimental phase*. For a detailed description of how to adapt the IDA Python code for different datasets, please refer to Supplementary Material S1.

#### IDA Deployment

The present IDA is hosted using PythonAnywhere (https://www.pythonanywhere.com), a Python-based online IDE and web hosting service that operates under a Freemium business model. With a free beginner account, every user is allowed to add one web application. In Table 1, we provide a short step-by-step tutorial on how to run an IDA on PythonAnywhere. Note that by default the web application’s URL includes the username and that permanent IDA deployment to PythonAnywhere requires users to log in at least once every three months to update the expiration date of the application. For an alternative hosting solution using Hugging Face Spaces, please refer to Supplementary Material S2.

**Table 1:** Step-by-Step Tutorial for Deploying a Python-Based Dash IDA to PythonAnywhere

(1)	Once registered with a beginner account and logged into PythonAnywhere, select the *Web* tab in the top bar and click on the *Add a new web app* button.
(2)	When prompted, select *Python* as the Python web framework as well as the required *Python version.*
(3)	Upload all required files (e.g., data files, code files) by navigating to the *Files* tab in the top bar and subsequently to *mysite* in the *Directories* tree on the left. Note that your Python-based Dash script should be named *app.py*. By default, a Python script named *flask_app.py* is contained in the remote PythonAnywhere folder, which should be removed. If existent, data directories defined within the Python-based Dash script must be adapted according to the folder structure present on PythonAnywhere.
(4)	Install all necessary dependencies by navigating to the *Consoles* tab in the top bar and selecting *Bash console.*
(5)	Navigate to *Web* and open the *WSGI configuration file* within the *Code* section. To enable communication between the web server and your IDA, change the last two lines of the file to (1) *from app import app*, (2) *application = app.server*
(6)	Navigate to *Web* and click on the *Reload* button. Your IDA should now be accessible.

In summary, the Dash-based IDA developed as Use Case 1 serves as an exemplary IDA for quantitative datasets, which, as detailed in Supplementary Material S1, may be readily adapted to different datasets. That said, the underlying dataset of interest must be in a reusable and preprocessed format, and IDA developers must provide information on which variables to include, which types of variables (e.g., categorical) shall be represented and analyzed, and how these variables should be named within the IDA. In case of large datasets, preprocessing the dataset for IDA deployment and providing the necessary information will likely be a somewhat time-consuming process. Moreover, different IDA components may be required to fulfil the IDA-specific purpose, for instance, different figure types and figure arrangements from the ones demonstrated here may be more appropriate for larger datasets. Finally, if of interest to the researchers, inferential statistics such as null hypothesis significance testing or the evaluation of Bayesian credible intervals could be incorporated in the IDA setup. Thus, while the possibilities for the development of IDAs are virtually limitless from a technical point of view, we believe that a clear vision of the aims and scope is a necessary precondition for the successful development of useful IDAs.

## Development of an Interactive Text and Sentiment Analysis Platform Using Gradio

### Introduction to Gradio

Gradio is an open-source Python library that allows for the online sharing and demonstration of machine learning models, data science workflows, and application programming interfaces (APIs). Specifically, Gradio enables users to provide access to algorithms and data analysis pipelines, allowing fellow researchers to test software-oriented research data in a playful manner. For example, the performance of a trained image classification algorithm may be demonstrated using a Gradio-based IDA by displaying the classification results for images provided by the IDA user. Gradio offers high customizability and user-friendliness with support for a large number of IDA components (e.g., images, texts, videos, chatbots) and extensive documentation. Moreover, the IDA layout customization is facilitated by the provision of prebuilt themes that can readily be accessed via gr.themes.*.

**Figure 4:**
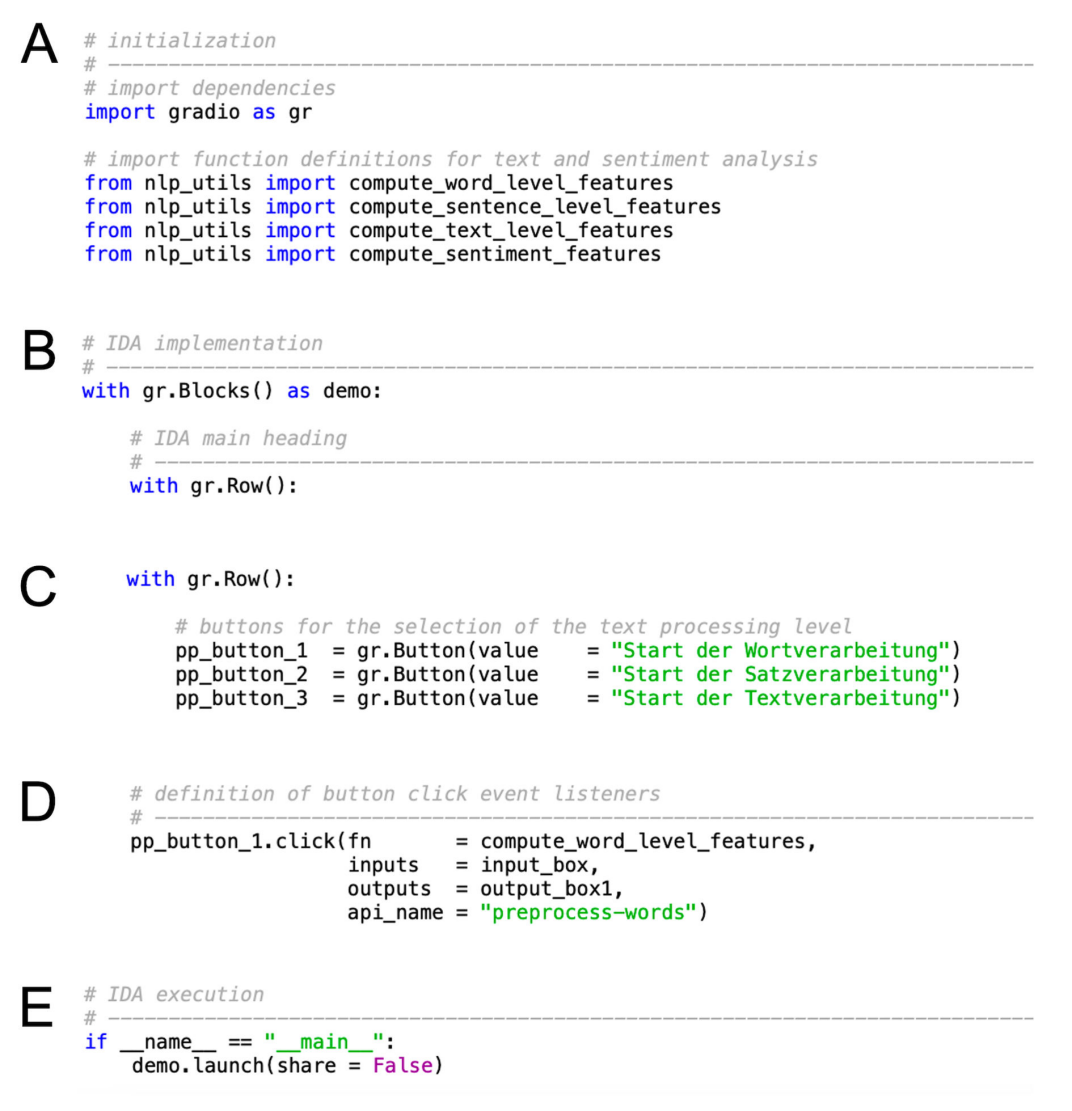
General Structure of Gradio-Based IDA Python Scripts

The general structure of a Python script that implements a Gradio-based IDA is comparably simple and easy to understand, allowing for fast IDA setup. As with Dash, a minimal Gradio IDA can be built with a few code lines. In principle, Gradio-based IDAs can be built by using either the *Interface class* or the *Blocks class*. In the following, we will focus on the latter since it offers a higher degree of flexibility and control. After the import of required dependencies (see Figure 4A), Blocks are initiated by calling the *Blocks class* inside a Python *with clause* (see Figure 4B).

In this way, a Blocks object is created and used as a *context*, which means that all components defined inside the *with clause* will be added to the IDA. By default, IDA components are arranged vertically in the order they are created. IDA components can be displayed side-by-side by using the layout element *gr.Row()* (see Figure 4B,C). Additional customization options include, for example, the specification of an IDA component’s size (e.g., *gr.Row().style(equal_height = True)* sets all IDA components that are displayed side by side to equal heights).

Interactivity of IDA components can be added by the definition of IDA component-specific *event listeners* (see Figure 4D). Event listeners are methods that execute a pre-defined function when triggered by a certain event, such as a button click. Each event listener requires a function definition and at least one IDA input and output component. When triggered by an event, the values of the IDA input component will be used by the event listener to execute the function definition for the generation of the value of the IDA output component. Notably, the number and order of the IDA input and output components specified within the event listener method must equal the number and order of function arguments and outputs.

A Gradio IDA is executed via the *launch* method, where the *share* argument specifies whether a public link for sharing purposes should be generated (see Figure 4E). This public link allows Gradio developers to provide other people with remote access to the IDA for a limited period of 72 hours, a feature that can be useful during development and testing of a Gradio-based IDA. Permanent hosting of Gradio IDAs can be easily implemented using Hugging Face Spaces (https://huggingface.co/spaces), a community-based platform for machine learning application sharing that operates under a Freemium business model. Hugging Face hosts IDAs on its own servers, while providing a sharable link and public access if desired. In addition, password-protected access options may be activated for sensitive ORD (see https://www.gradio.app/guides/sharing-your-app for further information). For a Gradio-based implementation of the data exploration board presented in the previous section, please refer to Supplementary Material S3.

### Use Case 2: An Interactive Text and Sentiment Analysis Platform

For the current use case, we consider the development of an IDA that demonstrates the nature and capabilities of a natural language processing (NLP) workflow. NLP aims to make computers able to understand and generate human language. Currently, NLP is routinely used across various application domains, such as chatbots, search engines, online translators, and product recommendations (Eisenstein, 2018; Raina & Krishnamurthy, 2022). Similarly, over the last decade, psychological research has increasingly employed NLP methods to make use of valuable data derived from routinely collected textual resources, such as transcripts of psychotherapy sessions (Shapira et al., 2021). The IDA we are concerned with relates to an NLP workflow that has recently been developed as part of a research project using textual data stemming from a psychotherapeutic Internet-based intervention (IBI) for depression. The respective IDA can be accessed directly using a standard web browser at https://huggingface.co/spaces/GradioIDA/gradio_demo.

Briefly, the previously mentioned research project examined the usefulness of linguistic features for the prediction of IBI treatment outcome, defined in terms of a statistically reliable and clinically relevant change in depression severity from pre- to post-treatment. To this end, letters from the IBI’s expressive writing tasks were analyzed by means of NLP workflows. At different stages of the IBI, participants were asked to write about three emotion-provoking topics concerning their depression. The raw, unstructured texts of 687 participants were then used for the computation of more than 800 non-affective and affective linguistic features. Notably, the linguistic features were derived from different processing units (i.e., single words, sentences, texts) and based on 12 different sentiment analysis dictionaries, each of which provided users with different information regarding the emotional content of the textual data.

#### Definition of the IDA’s Aims and Scope

**Figure 5:**
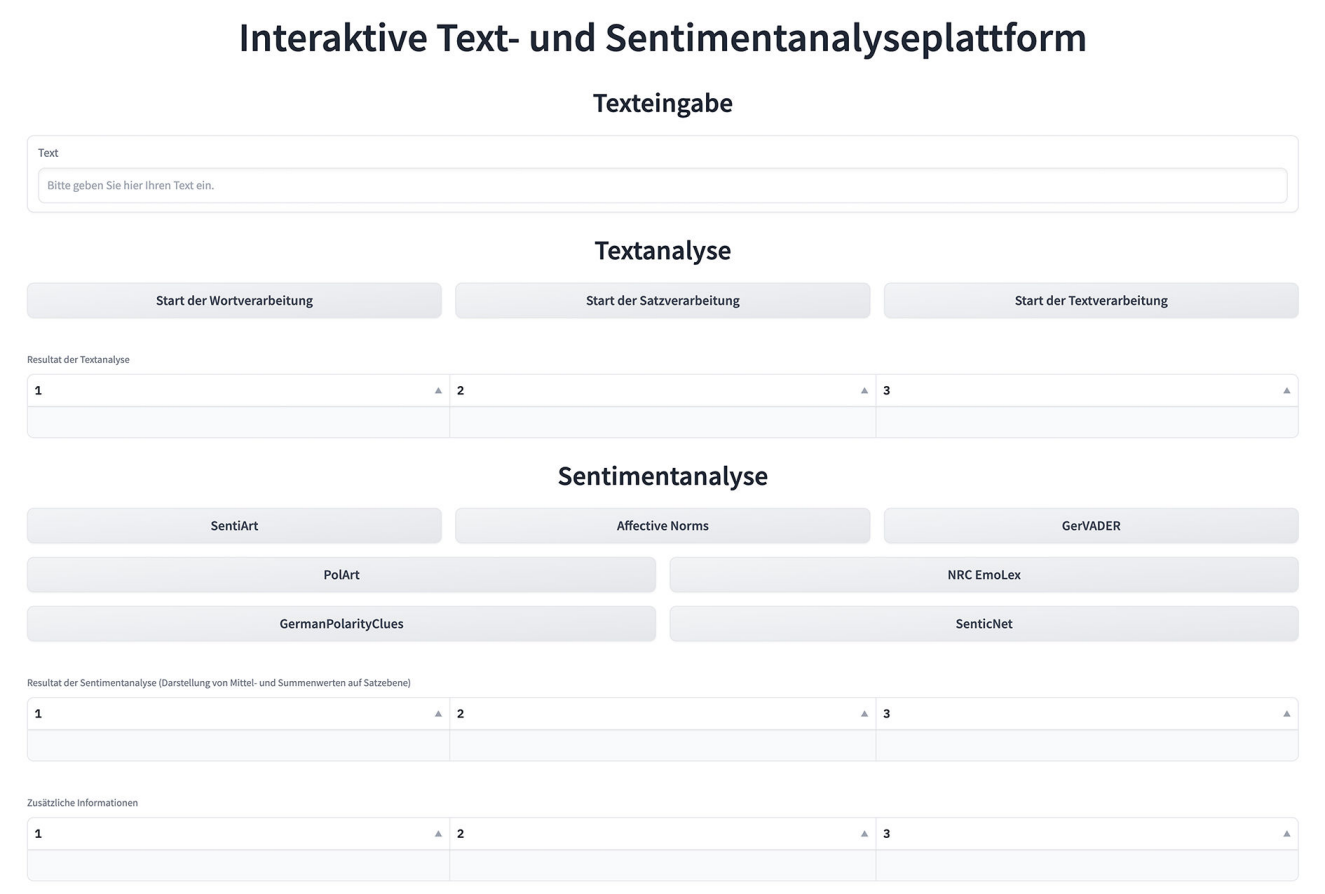
A Text and Sentiment Analysis Platform

The IDA developed for the current use case aimed to promote the understandability of and familiarization with the NLP workflows developed as part of the research project. We reasoned that in light of the large number of uniquely derived linguistic features and applied NLP methods, the understandability of the implemented NLP workflows may be limited, even if static codebooks and comprehensive data documentation files were provided. We thus intended to develop an IDA that allows interested readers to directly test parts of the described NLP workflows in an interactive and playful manner. To achieve our aims, we wanted IDA users to be able to provide their own text examples, choose between different types of text and sentiment analysis, and view the results (see Figure 5). The resulting IDA provides access and exploration opportunities for all methods developed for the purpose of text preprocessing, text analysis at different processing levels, and sentiment analysis frameworks used within the research project, allowing other researchers to quickly grasp whether a given workflow may be adaptable to their own research project. Note that due to the original textual data source, the IDA is restricted to the analysis of German texts. Also note that the selection of the sentiment analysis dictionaries included in the IDA was based on their open-source availability, their applicability to the German language, and their word coverage rather than a comparative evaluation of their relative merits and limitations.

#### IDA Programming and Testing

Based on the defined IDA’s aims and scope, the IDA layout was specified in terms of three sections, including user-provided textual input, text analysis at three different processing levels, and dictionary-based sentiment analysis. To enhance the readability of the Gradio Python script, function definitions required for text and sentiment analysis were delegated to a separate Python script called *nlp_utils.py*. Briefly, these function definitions implement basic text preprocessing and transformation steps (e.g., sentence boundary detection, tokenization, lemmatization, part-of-speech tagging), the detection of depression-relevant thematic words (e.g., sleep- or suicide-related words), the computation of affective linguistic features using seven sentiment analysis dictionaries (e.g., anger- or joy-relatedness), and the computation of linguistic features at the level of single words (e.g., word frequency, number of characters), single sentences (e.g., number of function words, number of pronouns), and the whole text (e.g., sentence-average and total number of verbs). For convenience, minor adaptations, including a different part-of-speech tagger and a different lemmatizer, were made to the original NLP workflows. Five originally applied sentiment analysis dictionaries could not be employed for the IDA due to their low word coverage or commercial restrictions on their use.

**Figure 6:**
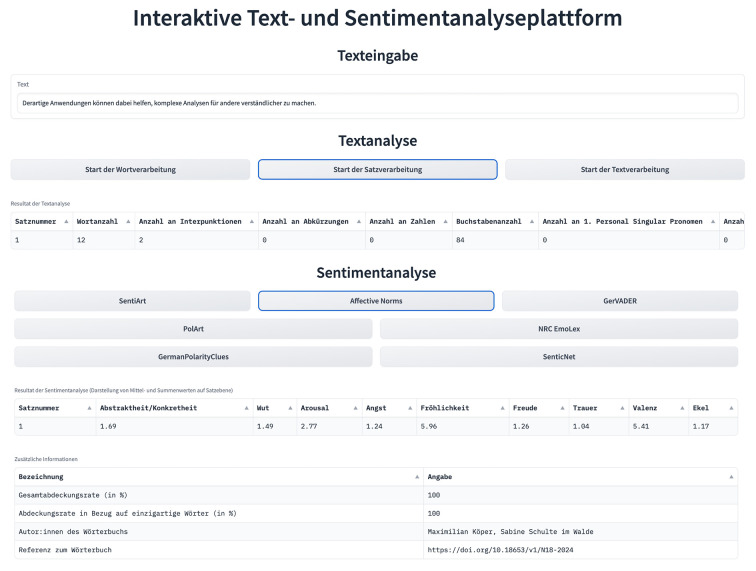
Exemplary Text and Sentiment Analysis Results

For the IDA layout, we used the default theme design and Gradio’s Markdown component *gr.Markdown()* in combination with HTML to implement section headings and adapt their position and font characteristics (e.g., using *<b></b>* within the Markdown component to print the text in bold). The text input field was added to the IDA by using the *gr.Textbox()* component, with the *label* and *placeholder* arguments defining the text displayed at the top of the box and within the box as long as no user-defined text is provided. Notably, no restrictions on the length of the provided text were included. IDA users are thus free to type in any German text, ranging from single words to whole paragraphs. All interactive buttons are implemented using the *gr.Button()* component. With the help of button-specific click event listeners, the respective function definitions for text and sentiment analysis are called automatically from *nlp_utils.py* once a button is activated. When loading the IDA, all output components such as the text analysis data table are empty by default (see Figure 5). Only when both an active text entry and a button click are registered is the respective IDA output component filled with results (see Figure 6). For the sentiment analysis section, both the sentiment analysis results and additional information on the selected sentiment analysis dictionary are displayed as IDA output components.

#### IDA Deployment

As mentioned above, the current IDA is hosted using Hugging Face Spaces. In general, there are three ways to deploy an IDA to Hugging Face Spaces: (1) using the terminal, (2) using a connection to a Git repository, and (3) using a browser. In Table 2, we provide a step-by-step tutorial for the latter. Note that in principle, Hugging Face Spaces allows users to run an unlimited number of models, datasets, spaces, and repositories, but if run on the default free hardware (CPU Basic), spaces will go to sleep if not visited for more than 48 hours.

**Table 2:** Step-by-Step Tutorial for Deploying a Gradio IDA to Hugging Face Spaces Using the Web Interface

(1)	Once registered with a free account and logged into Hugging Face, navigate to the *Spaces main page* (https://huggingface.co/spaces) and click on *Create new Space* on the right.
(2)	Choose the name of your space, select an optional license, set the visibility of your space (*private* or *public*), and select *Gradio* as the *Software Development Kit* (SDK).
(3)	Clicking *Create Space* will create a new repository. Here, navigate to *Files* in the top bar on the right and upload all required files in the desired directories by clicking on *+ Add files* on the right. The Python Gradio script should be named app.py. If existent, data directories defined within the script must be adapted according to the folder structure present on your space.
(4)	Create or upload a *requirements.txt* file with all required dependencies, i.e., Python libraries that must be installed to successfully deploy your IDA, in the main directory of your space.
(5)	Click on *App* in the top bar on the right and your IDA will be automatically built. In case of errors, you can click on *Logs* in the top bar on the left to obtain more information.

## Conclusion

The value of ORD lies in their reuse (Borgman, 2017; Quarati & Raffaghelli, 2022). However, the mere online availability does not seem to be sufficient for ORD reuse, and previous meta-scientific research has indicated that the underutilization of ORD is related to barriers at the level of the ORD themselves, potential reusers of ORD, and the broader academic ecosystem.

With the present work, we propose IDAs as innovative ORD supplements that may at least partially address these barriers. We demonstrated the exemplary use of two open-source Python libraries for IDA development using two psychological research use cases for which we anticipate reuse barriers due to their ORDs’ experimental or data-analytical complexity. In both cases, the IDA aims to foster the understandability of and familiarization with the respective ORD. We hope that the IDAs enable potential reusers to actively interact with the ORD in a joyful, user-friendly, and easily understandable manner.

Currently, the most common modes for sharing research data and code in psychology make use of static archiving platforms such as the Open Science Framework (Foster & Deardorff, 2017) or PsychArchives (Bosnjak, 2020) or collaborative development and version control solutions such as GitHub (Escamilla et al., 2022) or DataLad (Wagner & Hanke, 2023). Although these modes of providing ORD are clearly well-suited for distributing research artefacts accompanying research articles and have a strong potential to increase computational reproducibility in the field, they generally fall short of providing means to readily explore and interact with the ORD in a self-guided and interactive fashion. The IDA framework discussed here goes beyond these established solutions for ORD sharing, as, on the one hand, it requires the same rigor with respect to dataset documentation, long-term maintenance, and computational reproducibility assurance as do the existing ORD frameworks, while on the other hand, adding an interactive data exploration component as a low-threshold entry point for ORD reuse.

That said, it remains an open question whether IDAs actually promote research data reuse behaviors because the vast majority of meta-scientific ORD research thus far has focused on research data sharing (Joo et al., 2017) and ORD reuse remains difficult to assess (Pasquetto et al., 2019). Assuming for the moment that IDAs indeed foster ORD reuse and will be developed more routinely and on larger scales in the future, several limitations must be addressed. First, the permanent hosting of IDAs requires a sufficiently protected, professionally supported, large-scale, and easy-to-use infrastructure. Second, to the best of our knowledge, at present a one-size-fits-all IDA template does not exist, and IDAs must be designed and developed to address research project- and dataset-specific aims. In this regard, the implementation of IDAs requires additional time and effort, even if templates for IDA development are provided. However, this additional work is currently neither incentivized nor rewarded by the broader academic ecosystem. Third, researchers who are unfamiliar with programming might not feel sufficiently confident to dive into the development of IDAs.

These challenges could at least be partially addressed if large research institutions and academic publishing companies offered professional support systems for the development and permanent hosting of IDAs. For example, in an ideal world, research publishing companies could provide the infrastructure to transfer the ORD provided by academic researchers to suitable, possibly journal-specific IDAs, thereby adding genuine value to the research cycle. In this context, the issue of permanent hosting may be addressed by making permanent data and IDA hosting essential parts of research funding schemes or by capitalizing on novel dashboarding libraries that work independent of external hosting servers.

In summary, and the challenges discussed notwithstanding, we believe that IDAs have the potential to lower ORD reuse barriers and to contribute to a more transparent and sustainable research ecosystem in the information age.

## Supplementary Materials

The Supplementary Materials include the following items:

- The IDAs’ code and data as well as a codebook, a data preparation file, and additional information on the sentiment analysis dictionaries (see Usée et al., 2024S-a)
- A Supplementary Material file in which we provide additional information on case-specific adjustments for the Dash-based data exploration board (S1), on how to deploy Dash-based IDAs to Hugging Face (S2), and how to implement a data exploration board using Gradio (S3; see Usée et al., 2024S-b)

UséeF.
MelzigC. A.
OstwaldD.
 (2024S-a). Use it or lose it: Facilitating the use of interactive data apps in psychological research data sharing
[Research data, code, and additional materials]. PsychOpen. https://osf.io/y52p8/
10.5964/ejop.12811PMC1163869639678926

UséeF.
MelzigC. A.
OstwaldD.
 (2024S-b). Supplementary materials to "Use it or lose it: Facilitating the use of interactive data apps in psychological research data sharing"
[Additional information]. PsychOpen. 10.23668/psycharchives.15080
PMC1163869639678926

## Data Availability

The research data to replicate the findings of this study are publicly available (see Usée et al., 2024S-a).
